# Supplementary material for: Reducing FASN expression sensitizes acute myeloid leukemia cells to differentiation therapy
Source: Cell Death Differ. 2021 Mar 19;28(8):2465–81. doi: 10.1038/s41418-021-00768-1 (PMC8329134; doi:10.1038/s41418-021-00768-1)
Supplement: Supplementary file 1 — Supplementary Figure Legends [file 41418_2021_768_MOESM1_ESM.docx]

Supplementary Figure Legends:

**Supplementary Figure 1:** NB4 and HT93 APL cells were treated with ATRA at time points indicated. A-C. Flow cytometry analysis of CD11b surface expression of NB4 and HT93 cells upon ATRA treatment. A. Representative histograms. B. Median fluorescence intensity of 2 biological replicates. C. Percent of CD11b positive cells. D. qPCR analysis of *ELOVL6, SCD1* and *ACACA* transcript levels upon ATRA treatment. Values were normalized to the HMBS housekeeping gene. Results of at least three independent experiments are shown as n-fold regulation compared with control DMSO treated cells.

**Supplementary Figure 2:** EGCG treatment reduces FASN expression and increases autophagy activity upon ATRA treatment. NB4 cells were treated with different EGCG concentration (5µM, 10µM or 15µM) up to 3 days together with ATRA. A-B. Total lysate from ATRA- (A) and ATRA+ (B) treated cells were subjected to immunoblotting using anti-FASN. Total protein is shown as a loading control. The relative protein expressions were normalized to total protein. C-D. NBT reduction in ATRA-induced NB4 control and C75 treated cells. Scale 50µM C. Representative images of NBT assays. D. Quantification of the percentage of NBT^+^ cells. E. Flow cytometry analysis of CD11b surface expression of NB4 control, EGCG or EGCG/ATRA treated cells. F. NB4 cells were treated with EGCG at indicated concentrations in presence or absence of ATRA (1µM) and BafA1. Total lysate was subjected to immunoblotting using anti-p62 and anti-LC3B. Total protein is shown as loading control. G. NB4 cells were stably transfected with an mCherry-EGFP-LC3B construct and treated with different EGCG concentration together with ATRA for the indicated time points. The mCherry/EGFP ratio was determined by flow cytometry. H-I. NB4 parental cells were treated as in A and B. H. Non-fixed cells were stained with DAPI as a cell death marker. I. NB4 cell numbers were determined using trypan blue exclusion assay at day 0, 1, 2, 3 and 6.

**Supplementary Figure 3:** FASN depleted NB4 cells (shFASN_1/_2) were treated for 3 days with ATRA. A-B Cells were counted using typan blue exclusion at day 0, 1, 2, 3 and 6. A. Growth curve extracted from the cell counts. B. Doubling time of FASN depleted NB4 cells control or ATRA treated. C-D. γH2AX foci formation was analyzed by immunofluorescence upon ATRA treatment at day 1, 2 and 3. C. Representative pictures. Scale: 10µm. D. Quantification of foci per cells.

**Supplementary Figure 4:** NB4 parental cells were treated with either C75 at concentration 2.5µM, 5µM, 10µM or 20µM (A, C, E) or Orlistat 2.5µM or 5µM (B, D, F). A-B non fixed cells were stained with DAPI to measures cell death. C-D. NB4 cells were counted using typan blue exclusion at day 0, 1, 2, 3 and 6. E-F. Total cell lysates was subjected to immunoblotting using anti-FASN. Total protein is shown as loading control. G-H. FASN activity is inhibited by C75 and Orlistat. NB4 cells (0.5 x 105 cells/ml) were were labelled with 0.5 µCi/ml [14C]-Acetic Acid [American Radiolabeled Chemical Inc., St. Louis, MO, USA] for 6 hours in presence or absence of different concentrations of C75 and Orlistat and. Cells were harvested using 0.5 ml PBS and lipids were extracted using chloroform/methanol (3:1, v/v). The chloroform phase was evaporated under nitrogen and concentrated lipids were dissolved in 30 µl dichloromethane. Lipids were separed by thin layer chromatography over silicagel (SIL G/UV254) TLC plates (Macherey-Nagel, Oensingen, Switzerland) using hexane/diethyl ether/acetic acid (70:30:1, v/v) as mobile phase. Palmitic acid (PA) spots were identified using a standard [Sigma Aldrich, Basel, Switzerland] solution by exposing the TLC to Iodine vapour (G). The corresponding radiolabeled spots were visualised by autoradiography using a Fuji FLA-7000 PhosphoImager (Fujifilm, Dielsdorf, Switzerland) (H upper panel) and quantified using ImageQuant Software (H lower panel). The amount of PA formed in the presence of drugs was calculated as a percent of radioactivity incorporated into the control samples. Results are means of two independent experiments performed in triplicate.

**Supplementary Figure 5:** A. Schematic representation of TFEB activity. (B-C) Analysis of gene expression of *FASN*, *TFEB* and the CLEAR network in a TCGA-AML (B) and a Blood Spot normal human hematopoiesis with AML (C) data set.

**Supplementary Figure 6:** NB4 parental cells were treated for 3 days with 1µM ATRA and then subjected to (A) TFEB endogenous immunofluorescence (B) LAMP1 endogenous immunofluorescence and (C) Acridine Orange staining. A. Representative picture of TFEB localization in NB4 cells. Scale: 10µm. B. Representative picture of LAMP1 in NB4 cells. Scale: 10µm. C. Histogram representation of RED/GREEN ratio of NB4 cells treated with ATRA and stained with Acridine Orange.

**Supplementary Figure 7:** EGCG treatment accelerates TFEB translocation to the nucleus and improves lysosome biogenesis. NB4 and HT93 APL cells were treated for 1 to 3 days with ATRA in combination with indicated concentrations of EGCG. Cells were then subjected to (A) Western blot analysis for FASN and pmTOR(Ser2448), and (B) TFEB immunofluorescence. B. Representative pictures of TFEB in NB4 cells treated with ATRA and different concentrations of EGCG (5µM, 10µM and 15µM), Scale: 10µm. (C) Evaluation of *BECN1*, *GABARAP*, *STK4* and *WDR45* mRNA transcript levels was done by qPCR. Values were normalized to the HMBS housekeeping gene and are shown as n-fold regulation compared to control treated cells. Results are shown from at least two biological replicates.

**Supplementary Figure 8:** (A-B) LAMP1 endogenous immunofluorescence, and (C-E) Acridine Orange staining. A. Representative pictures of LAMP1 localization in NB4 cells treated with ATRA and different concentrations of EGCG. Scale: 10µm. B. LAMP1 punctae quantification in ATRA and EGCG treated cells at indicated time points. C. Histogram representation or the RED/GREEN ratio of NB4 cells treated with EGCG at indicated time points and concentrations. D. Representative histogram of NB4 cells treated with EGCG and ATRA at indicated time points and concentrations. E. Overton percentage positive quantification of the RED/GREEN ratio of NB4 cells treated with DMSO (upper panel) or ATRA (lower panel) at indicated time points and in combination with indicated EGCG concentrations.

**Supplementary Table 1:** Spearman Correlation coefficient of TCGA data set between FASN and CLEAR network expression.

**Supplementary Table 2:** Spearman Correlation coefficient of the bloodspot data set between FASN and CLEAR network expression.
