# Supplementary figures and images for: Reducing FASN expression sensitizes acute myeloid leukemia cells to differentiation therapy
Source: Cell Death Differ. 2021 Mar 19;28(8):2465–81. doi: 10.1038/s41418-021-00768-1 (PMC8329134; doi:10.1038/s41418-021-00768-1)

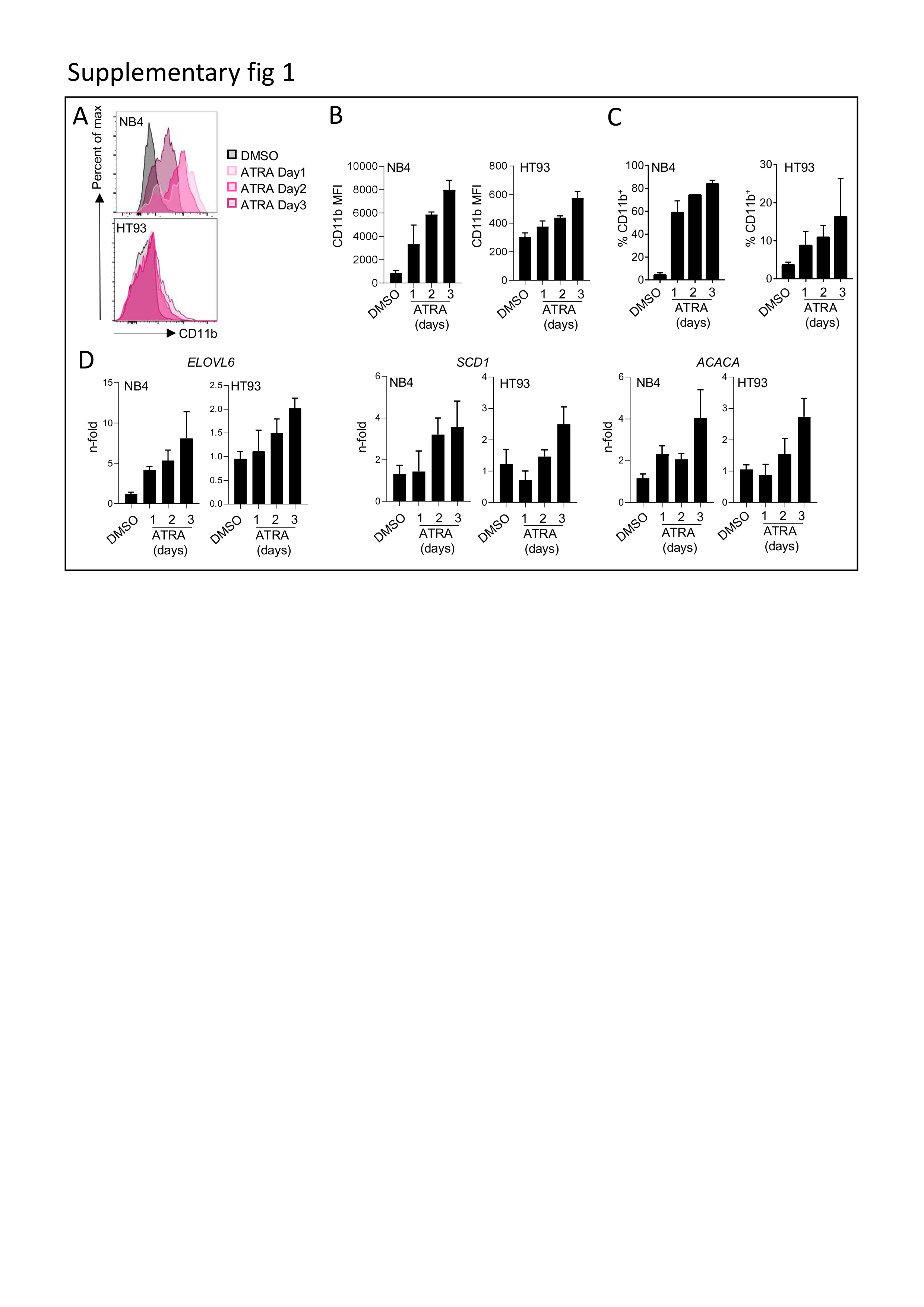

Supplement: Supplementary file 2 — Supplementary Figure 1 [file 41418_2021_768_MOESM2_ESM.tif]

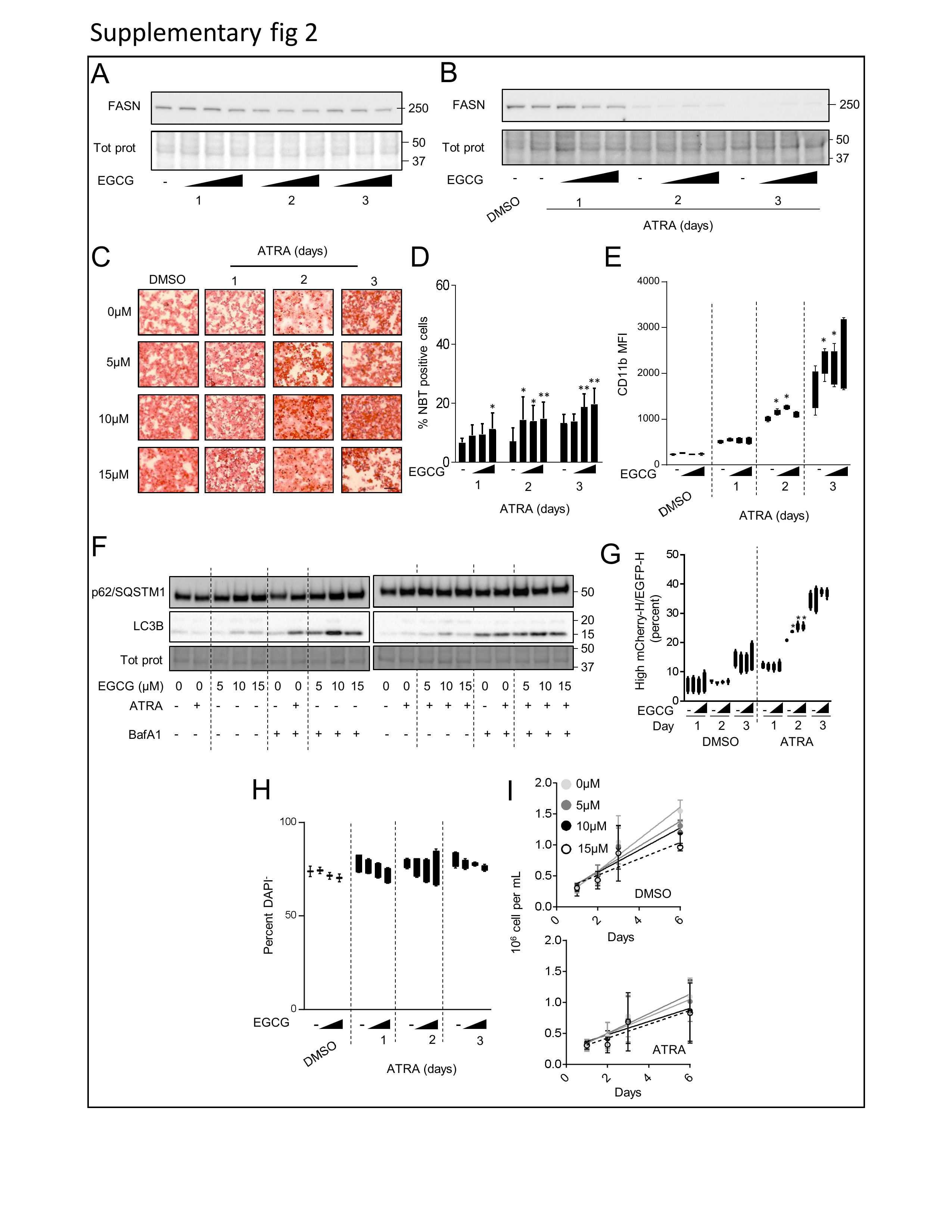

Supplement: Supplementary file 3 — Supplementary Figure 2 [file 41418_2021_768_MOESM3_ESM.tif]

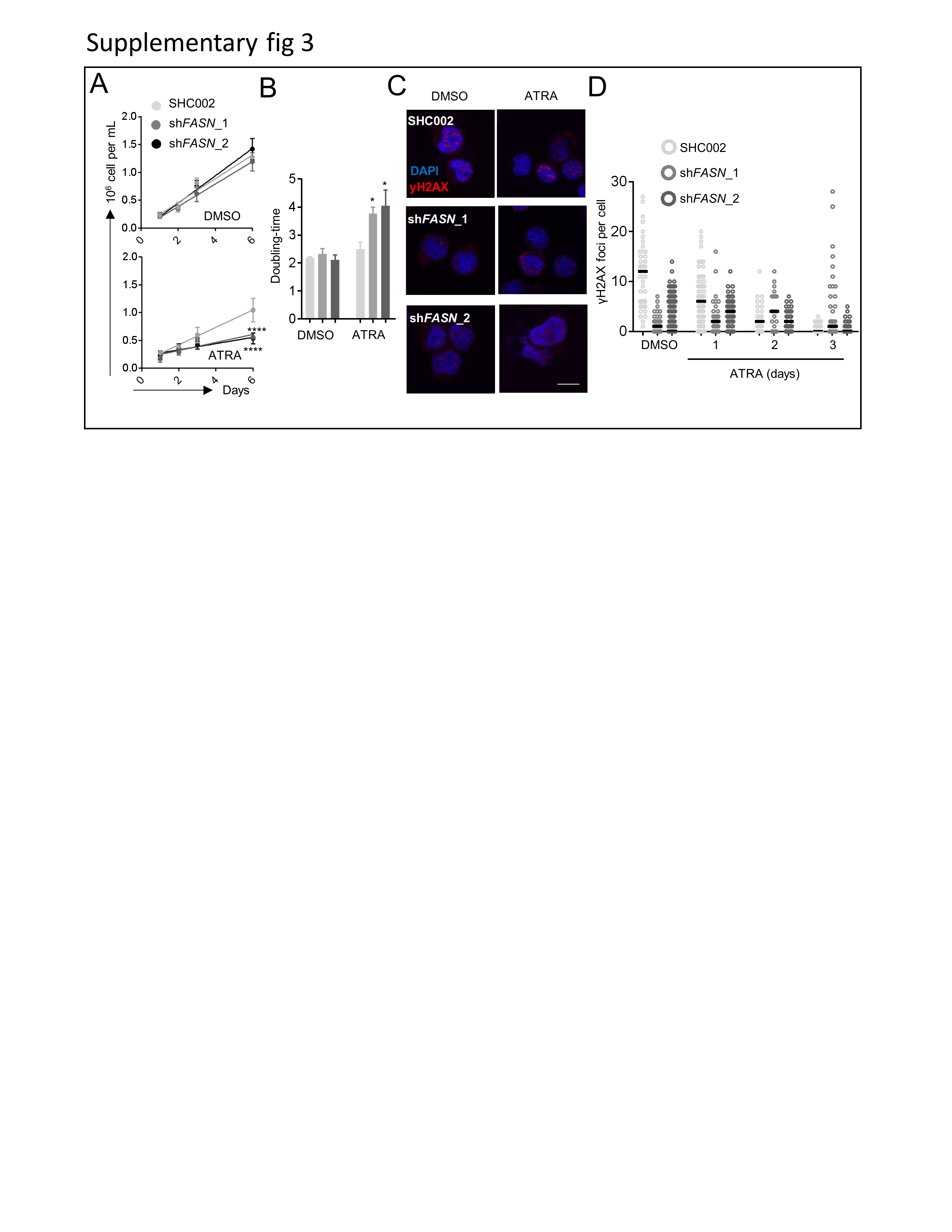

Supplement: Supplementary file 4 — Supplementary Figure 3 [file 41418_2021_768_MOESM4_ESM.tif]

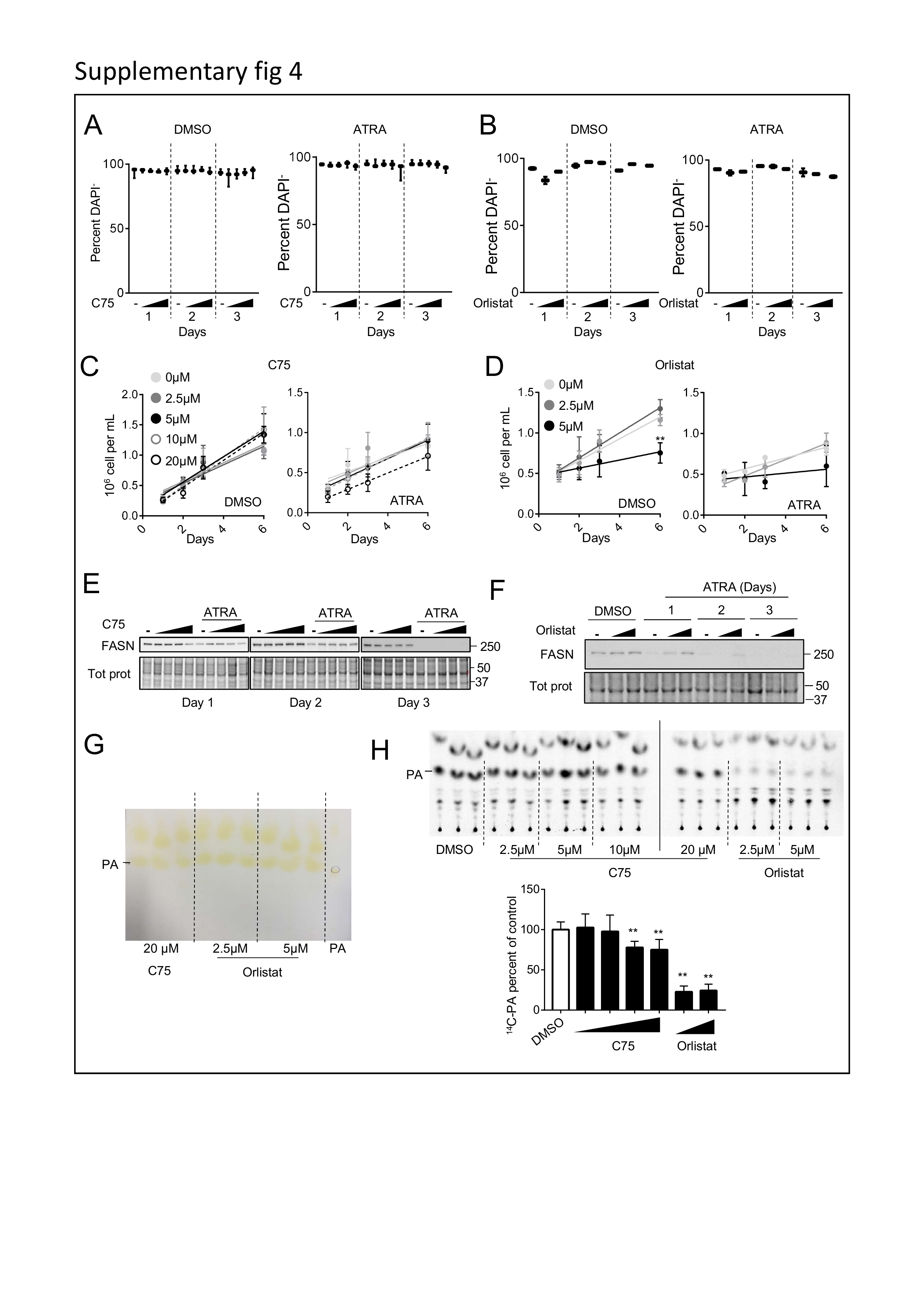

Supplement: Supplementary file 5 — Supplementary Figure 4 [file 41418_2021_768_MOESM5_ESM.tif]

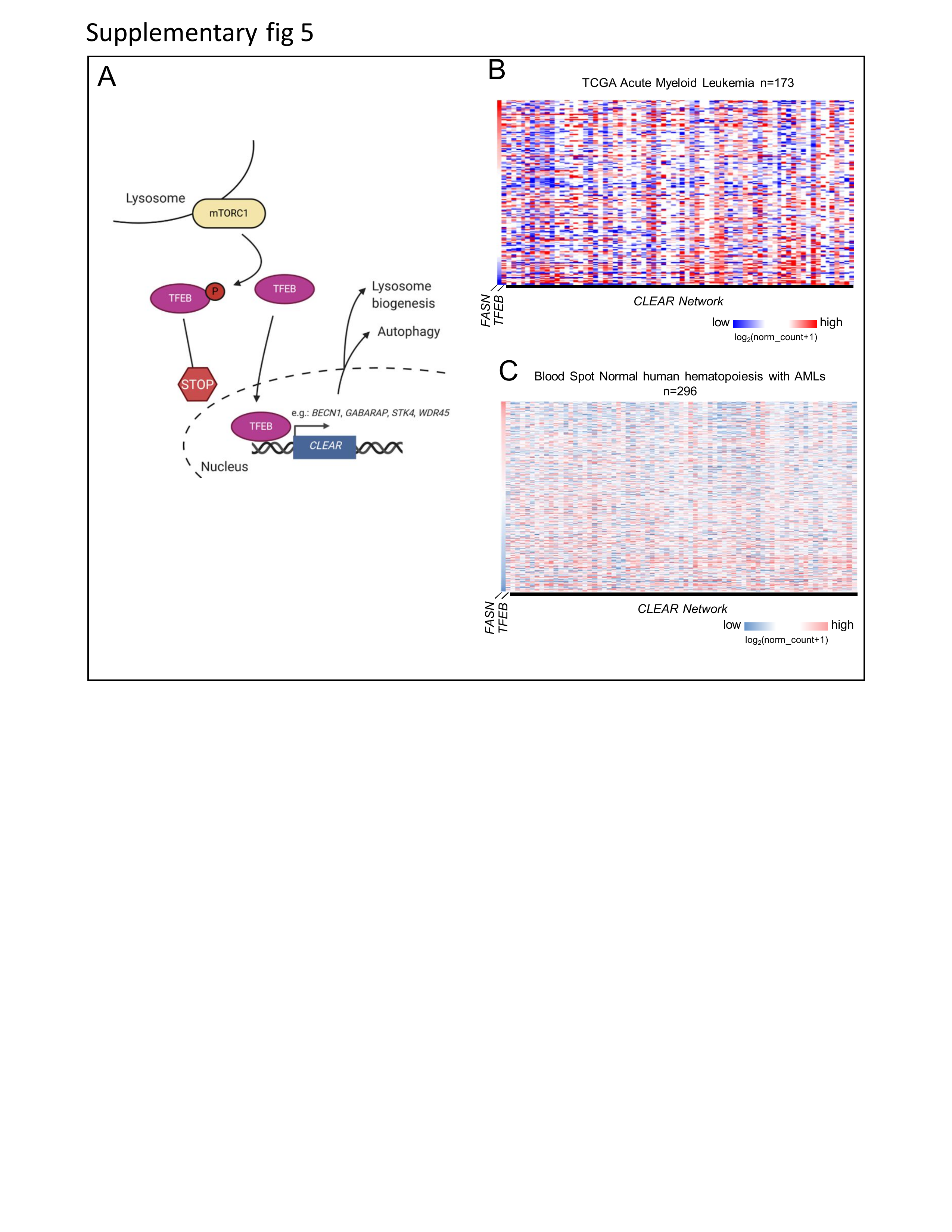

Supplement: Supplementary file 6 — Supplementary Figure 5 [file 41418_2021_768_MOESM6_ESM.tif]

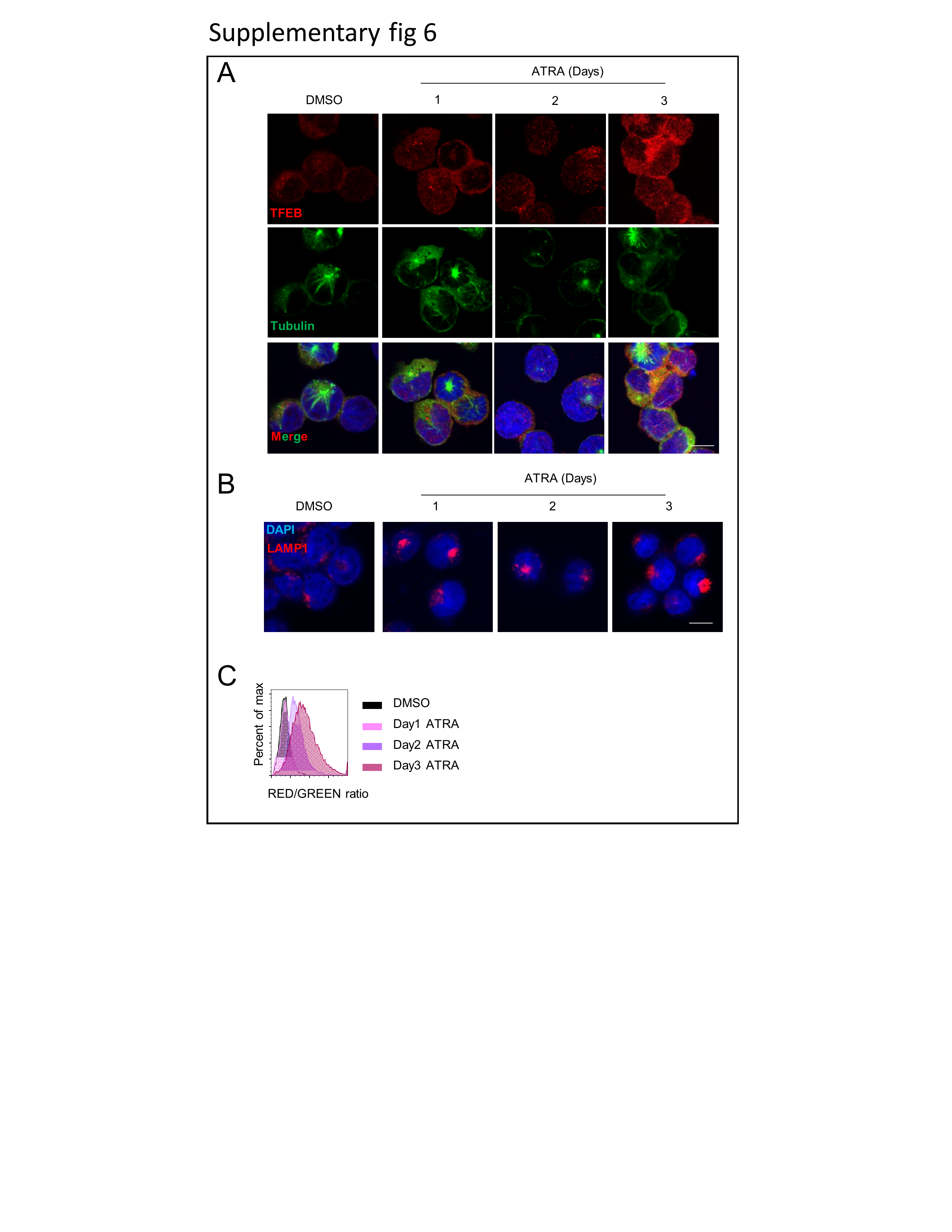

Supplement: Supplementary file 7 — Supplementary Figure 6 [file 41418_2021_768_MOESM7_ESM.tif]

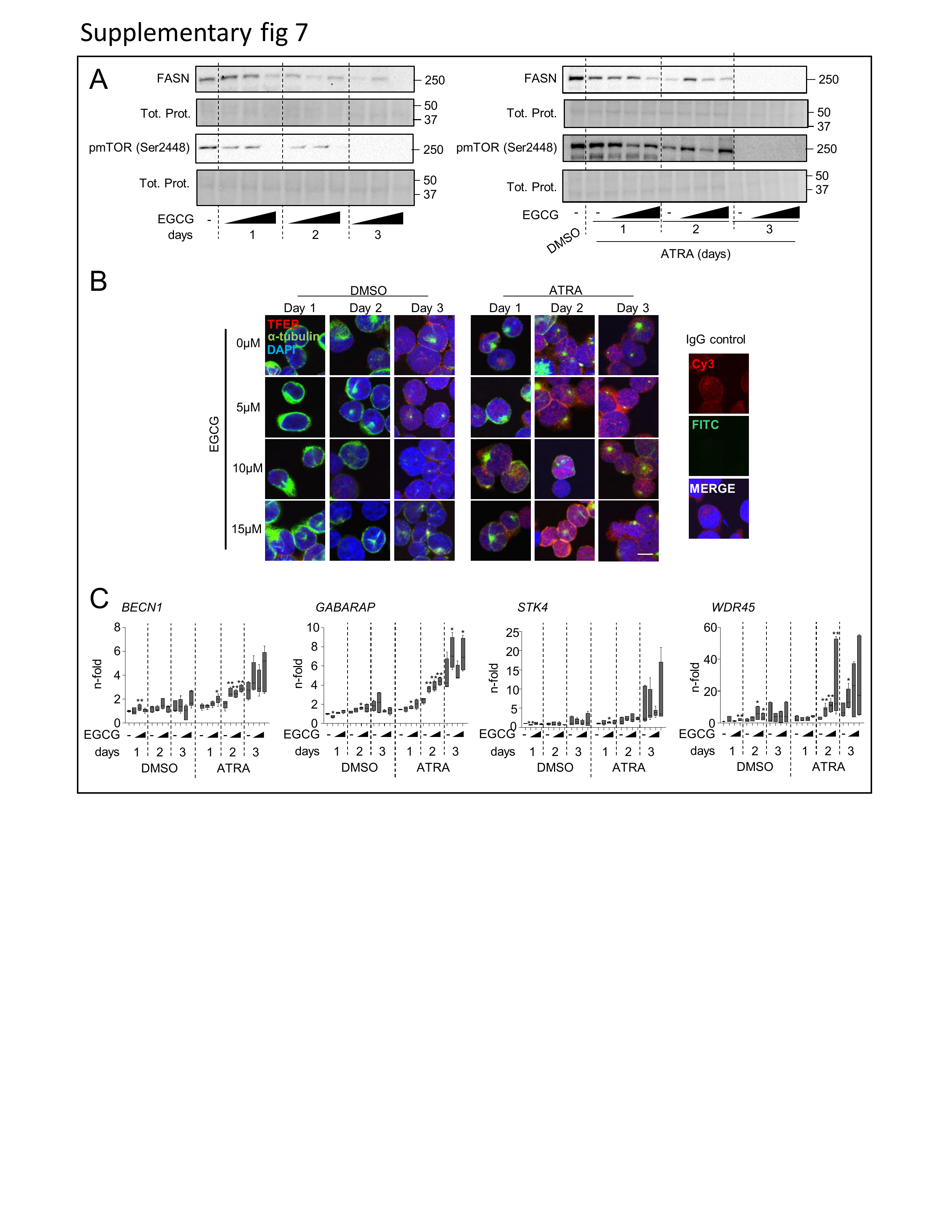

Supplement: Supplementary file 8 — Supplementary Figure 7 [file 41418_2021_768_MOESM8_ESM.tif]

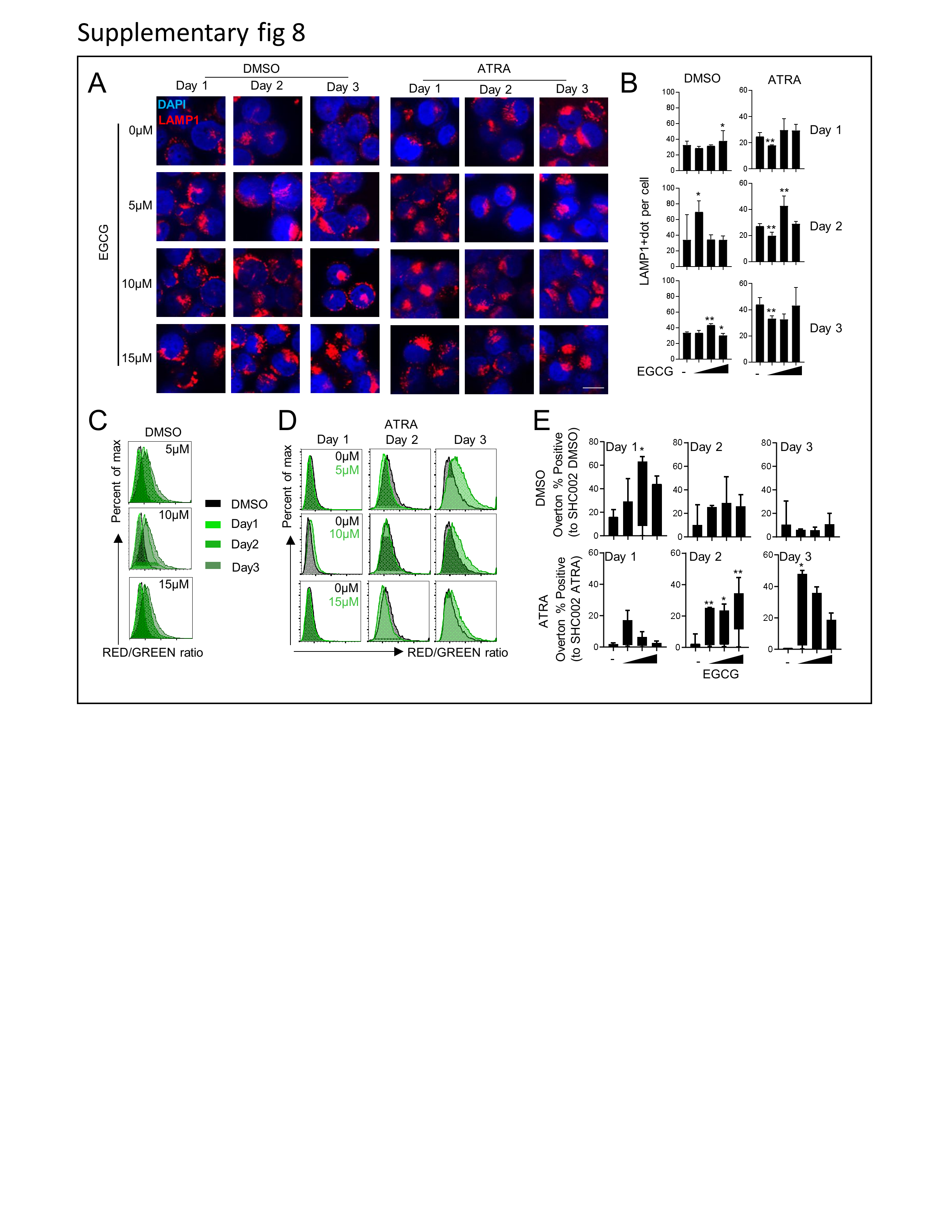

Supplement: Supplementary file 9 — Supplementary Figure 8 [file 41418_2021_768_MOESM9_ESM.tif]
